# Supplementary material for: Empirical methods for controlling false positives and estimating confidence in ChIP-Seq peaks
Source: BMC Bioinformatics. 2008 Dec 5;9:523. doi: 10.1186/1471-2105-9-523 (PMC2628906; doi:10.1186/1471-2105-9-523)
Supplement: Additional File 1 — A variety of html documents from the USeq web site detailing the available applications, their best usage, output file type descriptions, command line menus, etc. [file 1471-2105-9-523-S1.zip › USeqUserGuides/JavaDoc.zip!/JavaDoc/bioroot/strain/StrainPreference.html]

StrainPreference


|  |  |  |
| --- | --- | --- |
| **Overview** \| **Package** \| **Class** \| **Use** \| **Tree** \| **Deprecated** \| **Index** \| **Help** | |  |
| **PREV CLASS**   **NEXT CLASS** | **FRAMES**    **NO FRAMES**     **All Classes** |  |
| SUMMARY: NESTED \| FIELD \| CONSTR \| METHOD | DETAIL: FIELD \| CONSTR \| METHOD |  |


---


## bioroot.strain Class StrainPreference

```
java.lang.Object
  bioroot.Preference
      bioroot.strain.StrainPreference
```

---

``` public class StrainPreference extends Preference ```

**Author:**
:   nix

---

**Constructor Summary**

`StrainPreference()`

`StrainPreference(java.lang.String databaseConcat)`
  
          databaseConcat = visibility | orderBy| numberToDisplay | labGroups | features | strain types String[] separated by semi-colons


| **Method Summary** | |
| --- | --- |
| `java.lang.String[]` | `getStrainTypes()` |
| `java.lang.String` | `makeConcatination()`             Makes a visibility \| orderBy \| labGroups \| features \| strain types concat. |
| `void` | `setStrainTypes(java.lang.String[] strainTypes)` |

**Methods inherited from class bioroot.Preference**

`fetchNames, getFeatures, getLabGroups, getNumberToDisplay, getOrderBy, getVisibilities, getVisibility, isOrdered, setFeatures, setLabGroups, setNumberToDisplay, setOrderBy, setOrdered, setVisibility`

**Methods inherited from class java.lang.Object**

`equals, getClass, hashCode, notify, notifyAll, toString, wait, wait, wait`

**Constructor Detail**

### StrainPreference

```
public StrainPreference()
```

---


### StrainPreference

```
public StrainPreference(java.lang.String databaseConcat)
```

:   databaseConcat = visibility | orderBy| numberToDisplay | labGroups | features | strain types String[] separated by semi-colons


**Method Detail**

### makeConcatination

```
public java.lang.String makeConcatination()
```

:   Makes a visibility | orderBy | labGroups | features | strain types concat.

    :   **Overrides:**: `makeConcatination` in class `Preference`

---


### getStrainTypes

```
public java.lang.String[] getStrainTypes()
```

---


### setStrainTypes

```
public void setStrainTypes(java.lang.String[] strainTypes)
```


---


|  |  |  |
| --- | --- | --- |
| **Overview** \| **Package** \| **Class** \| **Use** \| **Tree** \| **Deprecated** \| **Index** \| **Help** | |  |
| **PREV CLASS**   **NEXT CLASS** | **FRAMES**    **NO FRAMES**     **All Classes** |  |
| SUMMARY: NESTED \| FIELD \| CONSTR \| METHOD | DETAIL: FIELD \| CONSTR \| METHOD |  |


---
